# Interethnic differences in pancreatic cancer incidence and risk factors: The Multiethnic Cohort

**DOI:** 10.1002/cam4.2209

**Published:** 2019-05-08

**Authors:** Brian Z. Huang, Daniel O. Stram, Loic Le Marchand, Christopher A. Haiman, Lynne R. Wilkens, Stephen J. Pandol, Zuo‐Feng Zhang, Kristine R. Monroe, Veronica Wendy Setiawan

**Affiliations:** ^1^ Department of Epidemiology UCLA Fielding School of Public Health Los Angeles California; ^2^ Department of Research & Evaluation Kaiser Permanente Southern California Pasadena California; ^3^ Department of Preventive Medicine, Keck School of Medicine University of Southern California Los Angeles California; ^4^ Epidemiology Program University of Hawaii Cancer Center Honolulu Hawaii; ^5^ Norris Comprehensive Cancer Center Los Angeles California; ^6^ Division of Gastroenterology, Department of Medicine Cedars‐Sinai Medical Center and Department of Veterans Affairs Los Angeles California

**Keywords:** cohort, epidemiology, ethnicity, incidence, minority, pancreatic cancer

## Abstract

While disparity in pancreatic cancer incidence between blacks and whites has been observed, few studies have examined disparity in other ethnic minorities. We evaluated variations in pancreatic cancer incidence and assessed the extent to which known risk factors account for differences in pancreatic cancer risk among African Americans, Native Hawaiians, Japanese Americans, Latino Americans, and European Americans in the Multiethnic Cohort Study. Risk factor data were obtained from the baseline questionnaire. Cox regression was used to estimate the relative risks (RRs) and 95% confidence intervals (CIs) for pancreatic cancer associated with risk factors and ethnicity. During an average 16.9‐year follow‐up, 1,532 incident pancreatic cancer cases were identified among 184,559 at‐risk participants. Family history of pancreatic cancer (RR 1.97, 95% CI 1.50‐2.58), diabetes (RR 1.32, 95% CI 1.14‐1.54), body mass index ≥30 kg/m^2^ (RR 1.25, 95% CI 1.08‐1.46), current smoking (<20 pack‐years RR 1.43, 95% CI 1.19‐1.73; ≥20 pack‐years RR 1.76, 95% CI 1.46‐2.12), and red meat intake (RR 1.17, 95% CI 1.00‐1.36) were associated with pancreatic cancer. After adjustment for these risk factors, Native Hawaiians (RR 1.60, 95% CI 1.30‐1.98), Japanese Americans (RR 1.33, 95% CI 1.15‐1.54), and African Americans (RR 1.20, 95% CI 1.01‐1.42), but not Latino Americans (RR 0.90, 95% CI 0.76‐1.07), had a higher risk of pancreatic cancer compared to European Americans. Interethnic differences in pancreatic cancer risk are not fully explained by differences in the distribution of known risk factors. The greater risks in Native Hawaiians and Japanese Americans are new findings and elucidating the causes of these high rates may improve our understanding and prevention of pancreatic cancer.

## 1 INTRODUCTION

Pancreatic cancer is one of the deadliest malignancies in the United States. It has a 5‐year survival rate of only 8% and is the third most common cancer‐related death among men and women.^1^ By 2030, pancreatic cancer is estimated to surpass colorectal cancer to become the second leading cause of cancer mortality.^2^ Given these circumstances, a better understanding of its risk factors is imperative for preventing pancreatic cancer occurrence.

Ethnic differences in pancreatic cancer incidence have been investigated in several previous settings.^3^ Most prior literature has focused on the higher rates of pancreatic cancer in African Americans compared to whites,^4^, ^5^ with some research suggesting that this disparity may be partially explained by the greater prevalence of smoking, diabetes, and obesity among African Americans.^4^, ^6^ However, only a few studies have assessed other ethnic minorities, which have observed that Hispanics and Asians/Pacific Islanders have lower incidence rates of pancreatic cancer compared to whites and African Americans.^7^, ^8^ Additionally, no previous study has closely evaluated the relationships between known risk factors and pancreatic cancer across multiple ethnic minorities. Thus, there is limited information as to whether risk factors identified in past literature explain varying degrees of pancreatic cancer incidence between racial/ethnic subpopulations.

The purpose of this study was to investigate differences in pancreatic cancer incidence across African Americans, Native Hawaiians, Japanese Americans, Latino Americans, and European Americans in the Multiethnic Cohort (MEC). In particular, we sought to assess the extent to which known risk factors account for these potential differences and whether the influence of specific risk factors varies by race/ethnicity. Understanding these differences may allow us to better elucidate pancreatic cancer etiology across ethnic groups and establish more targeted prevention strategies.

## 2 METHODS

### 2.1 Study population

The MEC was established in 1993‐1996 to investigate cancer etiology. It is comprised of >215,000 participants aged 45‐75 at entry recruited from Los Angeles County and Hawaii. The five main ethnic groups are European American, African American, Latino American, Japanese American, and Native Hawaiian.^9^ All participants completed a self‐administered baseline questionnaire, which included information on demographics, medical conditions, family history of cancer, and lifestyle factors. Individuals were excluded from this study if they were not in the five main race/ethnicity groups (n = 13,987), had a prior pancreatic cancer diagnosis (n = 56), or were missing information on diet, diabetes, body mass index (BMI), and smoking (n = 12,957). Smokers with missing pack‐years data were further excluded (n = 4,091).

### 2.2 Exposure assessment

Exposure information was obtained from the baseline questionnaire. Participants were asked to self‐report their race/ethnicity, height, weight, diabetes history, family history of cancer (first‐degree relative), and frequency and duration of cigarette smoking. Smoking was evaluated as overall status (never, past, current) and with pack‐year information (never, past with <20 pack‐years, past with ≥20 pack‐years, current with <20 pack‐years, current with ≥20 pack‐years). Height and weight measurements were used to calculate BMI (kg/m^2^). Alcohol use (g/day) and red meat (processed and nonprocessed) intake (g/kcal/day) in the year prior to cohort entry were assessed through a food frequency questionnaire validated for a multiethnic population.^10^

### 2.3 Outcome

Participants were followed from cohort entry to pancreatic cancer diagnosis, death, or end of follow‐up (31 December 2013). Annual linkages with the statewide Surveillance, Epidemiology and End Results (SEER) registries of Hawaii and California were used to identify incident cases of pancreatic cancer (ICD‐O‐3 codes C25.0‐C29.9). Death dates for right‐censoring were ascertained using linkages with death certificate files for Hawaii and California and the National Death Index.

### 2.4 Statistical analyses

Pancreatic cancer incidence rates, left truncated at age 45 and age‐standardized using the United States 2000 standard population, were calculated for each race/ethnicity group. Race/ethnicity‐specific incidence rates were compared to the incidence rates for European Americans by testing the standardized rate ratios. Cox models with time since cohort entry as the time metric were used to assess the influence of race/ethnicity and risk factors on pancreatic cancer incidence. Minimally adjusted models, with age and sex as strata variables and race/ethnicity as a covariate, were fitted for each exposure. We also ran fully adjusted models which included age and sex as strata variables and race/ethnicity, family history of pancreatic cancer, diabetes, BMI (<25, 25‐30, ≥30 kg/m^2^), smoking with pack‐year information (never, past with <20 pack‐years, past with ≥20 pack‐years, current with <20 pack‐years, current with ≥20 pack‐years), alcohol use (none, <24, 24‐48, >48 g/day), and red meat intake (examined as quartiles) as covariates. Trends were examined using a model that treated the exposure as a continuous variable coded as consecutive numbers (eg, 1, 2, 3). To account for potential residual confounding by smoking, we ran models incorporating more detailed smoking variables,^11^ including current status, number of cigarettes smoked, smoking duration, and time since quitting. As this further adjustment did not change the results, only the results from the original models are presented. Race/ethnicity‐stratified analyses were performed to evaluate the relationships between risk factors and pancreatic cancer in each subpopulation. Tests of heterogeneity were conducted using models with cross‐product terms for each risk factor and race/ethnicity.

Population attributable fractions (PAF) were calculated for risk factors positively associated with pancreatic cancer using the method developed for cohort studies.^12^ This method calculates the PAF and 95% confidence intervals for various risk behavior modifications across specified follow‐up time intervals, while accounting for other risk factors and mortality as a competing risk. We calculated the PAFs associated with modifications in risk factors including BMI (≥25 to <25, ≥30 to <25 kg/m^2^), smoking (current to never), diabetes (yes to no), and red meat intake (second/third/fourth quartiles to first quartile). We also calculated PAFs for all of the aforementioned risk factors combined. Population attributable fractions were calculated for the entire cohort as well as for each racial/ethnic group over a 20‐year follow‐up period.

Schoenfeld residuals were used to verify the proportional hazards assumption and Martingale and deviance residuals were assessed to determine model fit.^13^ Analyses were conducted using SAS 9.3 (Cary, NC) and reported *P*‐values are two‐sided.

## 3 RESULTS

This study consisted of 184,559 at‐risk individuals (100,969 females and 83,590 males). The largest racial/ethnic group was Japanese Americans (29.0%), followed by European Americans (25.1%), Latino Americans (22.0%), African Americans (16.7%), and Native Hawaiians (7.3%). The average age at cohort entry was 59.9 (standard deviation 8.8). Approximately half of the participants were nonsmokers (44.9%) and non‐alcohol users (51.2%), while nearly 60% of the cohort was either overweight or obese. In addition, 11.7% of participants had diabetes while only 1.7% had a family history of pancreatic cancer (Table 1).

**Table 1:** Baseline characteristics of Multiethnic Cohort Study participants from 1993 to 2013

Characteristic	Total (N = 184,559)		European American (N = 46,356)		African American (N = 30,731)		Native Hawaiian (N = 13,412)		Japanese American (N = 53,429)		Latino American (N = 40,631)	
	N	%	N	%	N	%	N	%	N	%	N	%
Age at baseline (y)^a^	59.9 (8.8)		58.9 (9.1)		61.0 (9.0)		56.4 (8.6)		61.0 (9.1)		59.7 (7.8)	
Age group at baseline												
<50	31,095	16.9	9,454	20.4	4,452	14.5	3,791	28.3	8,438	15.8	4,960	12.2
50‐54	27,673	15.0	7,938	17.1	4,283	13.9	2,683	20.0	7,040	13.2	5,729	14.1
55‐59	29,370	15.9	6,985	15.1	4,548	14.8	2,110	15.7	6,794	12.7	8,933	22.0
60‐64	31,744	17.2	7,144	15.4	4,296	14.0	1,961	14.6	8,892	16.6	9,451	23.3
65‐69	32,409	17.6	7,190	15.5	6,366	20.7	1,601	11.9	10,614	19.9	6,638	16.3
≥70	32,268	17.5	7,645	16.5	6,786	22.1	1,266	9.4	11,651	21.8	4,920	12.1
Sex												
Male	83,590	45.3	21,434	46.2	11,235	36.6	5,883	43.9	25,336	47.4	19,702	48.5
Female	100,969	54.7	24,922	53.8	19,496	63.4	7,529	56.1	28,093	52.6	20,929	51.5
Family history of pancreatic cancer	3,175	1.7	800	1.7	357	1.2	197	1.5	1,250	2.3	571	1.4
Diabetes	21,563	11.7	2,741	5.9	4,822	15.7	1,988	14.8	5,626	10.5	6,386	15.7
Body mass index (kg/m^2^)												
<25	76,973	41.7	21,356	46.1	8,295	27.0	3,578	26.7	32,208	60.3	11,536	28.4
25‐30	70,865	38.4	16,854	36.4	12,580	40.9	5,037	37.6	17,471	32.7	18,923	46.6
≥30	36,721	19.9	8,146	17.6	9,856	32.1	4,797	35.8	3,750	7.0	10,172	25.0
Smoking status												
Never	82,852	44.9	18,229	39.3	11,910	38.8	5,305	39.6	26,991	50.5	20,417	50.2
Past	72,086	39.1	20,499	44.2	11,907	38.8	5,082	37.9	20,152	37.7	14,446	35.6
Current	29,621	16.1	7,628	16.5	6,914	22.5	3,025	22.6	6,286	11.8	5,768	14.2
Pack‐years^a^	10.2 (15.0)		13.5 (17.6)		10.0 (13.4)		12.2 (15.8)		9.8 (14.8)		6.4 (11.5)	
Smoking status, with pack‐year information												
Never	82,852	44.9	18,229	39.3	11,910	38.8	5,305	39.6	26,991	50.5	20,417	50.3
Past <20 pack‐years	53,370	28.9	13,702	29.6	9,431	30.7	3,648	27.2	14,253	26.7	12,336	30.4
Past ≥20 pack‐years	18,716	10.1	6,797	14.7	2,476	8.1	1,434	10.7	5,899	11.0	2,110	5.2
Current <20 pack‐years	15,960	8.7	2,778	6.0	4,528	14.7	1,556	11.6	3,006	5.6	4,092	10.1
Current ≥20 pack‐years	13,661	7.4	4,850	10.5	2,386	7.8	1,469	11.0	3,280	6.1	1,676	4.1
Alcohol use (g/day)												
None	94,446	51.2	16,124	34.8	17,187	55.9	7,197	53.7	33,280	62.3	20,658	50.8
<24	69,009	37.4	21,379	46.1	10,858	35.3	4,731	35.3	15,715	29.4	16,326	40.2
24‐48	13,158	7.1	5,573	12.0	1,539	5.0	912	6.8	3,015	5.6	2,119	5.2
>48	7,946	4.3	3,280	7.1	1,147	3.7	572	4.3	1,419	2.7	1,528	3.8
Red meat intake (g/kcal/day)												
Q1 (0‐14.1)	46,140	25.0	14,903	32.1	7,681	25.0	2,181	16.3	13,051	24.4	8,324	20.5
Q2 (14.1‐23.9)	46,139	25.0	12,038	26.0	7,186	23.4	3,061	22.8	14,335	26.8	9,519	23.4
Q3 (23.9‐35.2)	46,140	25.0	10,428	22.5	7,282	23.7	3,809	28.4	14,350	26.9	10,271	25.3
Q4 (35.2‐216.5)	46,140	25.0	8,987	19.4	8,582	27.9	4,361	32.5	11,693	21.9	12,517	30.8
Education												
≤12 y	79,978	43.3	12,149	26.2	12,312	40.1	7,034	52.5	21,173	39.6	27,310	67.2
Some college/vocational	54,499	29.5	14,584	31.5	11,363	37.0	4,000	29.8	15,781	29.5	8,771	21.6
College graduate	49,311	26.7	19,537	42.1	6,919	22.5	2,344	17.5	16,344	30.6	4,167	10.3
Missing	771	0.4	86	0.2	137	0.5	34	0.3	131	0.3	383	0.9

All tests comparing distribution of risk factors across race/ethnicity groups had *P* < 0.0001.

a Mean (standard deviation).

The prevalence of risk factors differed across racial/ethnic groups. Family history of pancreatic cancer was slightly more prevalent in Japanese Americans (2.3%) compared to the other subpopulations. Diabetes was less common in European Americans (5.9%) and more common in Japanese Americans (10.5%), Native Hawaiians (14.8%), African Americans (15.7%), and Latino Americans (15.7%). African Americans, Native Hawaiians, and Latino Americans were more likely to have BMI ≥25 kg/m^2^. Current smoking was more prevalent in Native Hawaiians (22.6%) and African Americans (22.5%), and less prevalent among Japanese Americans (11.8%) and Latino Americans (14.2%). Alcohol use was more commonly observed in European Americans (65.2%), while red meat intake (highest quartile) was greater among African Americans (27.9%), Latino Americans (30.8%), and Native Hawaiians (32.5%) (Table 1).

There were 1,532 incident cases of pancreatic cancer over an average follow‐up period of 16.9 years. Age‐standardized pancreatic cancer incidence rates were lowest in European Americans and highest in Native Hawaiians. Compared to European Americans (41.3 per 100,000 person‐years), Native Hawaiians (73.4), Japanese Americans (56.8), and African Americans (52.7) all had higher rates (*P* < 0.01). There was no difference in the incidence rates between Latino Americans (42.0) and European Americans (*P* = 0.87) (Table 2).

**Table 2:** Age‐adjusted incidence rates of pancreatic cancer, by race/ethnicity

Race/ethnicity	N	Cases	Person‐years	Age‐adjusted incidence rate^a^	*P* b
European American	46,356	306	784,877.9	41.3	
African American	30,731	277	488,746.5	52.7	<0.01
Native Hawaiian	13,412	128	221,361.6	73.4	<0.001
Japanese American	53,429	545	922,177.0	56.8	<0.0001
Latino American	40,631	276	697,674.8	42.0	0.87

a Incidence rate per 100,000 person‐years, age‐standardized to US Census 2000 standard population, left truncated at age 45.

b From a test comparing incidence rates to European Americans based on standardized rate ratios.

We also detected an increased risk of pancreatic cancer for Native Hawaiians, Japanese Americans, and African Americans in our Cox models (Table 3). When only adjusting for age and sex, Native Hawaiians (RR 1.75, 95% CI 1.42‐2.15), Japanese Americans (RR 1.32, 95% CI 1.15‐1.52), and African Americans (RR 1.34, 95% CI 1.13‐1.57) were all at greater risk for pancreatic cancer compared to European Americans. These risks remained elevated after adjusting for family history of pancreatic cancer, diabetes, BMI, smoking, alcohol, and red meat intake. Native Hawaiians had a 60% increased risk (RR 1.60, 95% CI 1.30‐1.98), Japanese Americans had a 33% increased risk (RR 1.33, 95% CI 1.15‐1.54), and African Americans had a 20% increased risk (RR 1.20, 95% CI 1.01‐1.42) compared to European Americans (Table 3). Native Hawaiians also had a higher risk compared to African Americans (*P* = 0.01), but not compared to Japanese Americans (*P* = 0.06). The risks for Japanese Americans and African Americans were not different from each other (*P* = 0.21).

**Table 3:** Associations of race/ethnicity and various risk factors and pancreatic cancer risk

Risk factor	Cases	Person‐years	Minimally adjusted^a^ RR (95% CI)	Fully adjusted^b^ RR (95% CI)
Race/ethnicity				
European American	306	784,877.9	1 (ref)	1 (ref)
African American	277	488,746.5	1.34 (1.13‐1.57)	1.20 (1.01‐1.42)
Native Hawaiian	128	221,361.6	1.75 (1.42‐2.15)	1.60 (1.30‐1.98)
Japanese American	545	922,177.0	1.32 (1.15‐1.52)	1.33 (1.15‐1.54)
Latino American	276	697,674.8	0.95 (0.81‐1.12)	0.90 (0.76‐1.07)
Family history of pancreatic cancer				
No	1,478	3,060,983.6	1 (ref)	1 (ref)
Yes	54	53,854.1	1.93 (1.47‐2.53)	1.97 (1.50‐2.58)
Diabetes				
No	1,316	2,808,732.7	1 (ref)	1 (ref)
Yes	216	306,105.0	1.37 (1.18‐1.58)	1.32 (1.14‐1.54)
Body mass index (kg/m^2^)				
<25	625	1,299,492.5	1 (ref)	1 (ref)
25‐30	599	1,203,354.1	1.09 (0.97‐1.23)	1.09 (0.97‐1.23)
≥30	308	611,991.0	1.27 (1.10‐1.47)	1.25 (1.08‐1.46)
p_trend_ c			<0.01	0.01
Smoking status, with pack‐year information				
Never	679	1,459,583.7	1 (ref)	1 (ref)
Past <20 pack‐years	414	908,445.2	1.00 (0.88‐1.13)	0.99 (0.87‐1.12)
Past ≥20 pack‐years	152	279,328.2	1.02 (0.85‐1.22)	0.97 (0.81‐1.17)
Current <20 pack‐years	140	264,554.3	1.44 (1.20‐1.74)	1.43 (1.19‐1.73)
Current ≥20 pack‐years	147	202,926.3	1.77 (1.47‐2.13)	1.76 (1.46‐2.12)
p_trend_ c			<0.0001	<0.0001
Alcohol use (g/day)				
None	820	1,570,115.6	1 (ref)	1 (ref)
<24	544	1,197,773.7	0.97 (0.87‐1.09)	0.98 (0.88‐1.11)
24‐48	108	221,918.6	1.03 (0.83‐1.26)	1.00 (0.81‐1.24)
>48	60	125,029.8	0.99 (0.76‐1.30)	0.95 (0.73‐1.25)
p_trend_ c			0.94	0.66
Red meat intake (g/kcal/day)				
Q1 (0‐14.1)	347	790,241.8	1 (ref)	1 (ref)
Q2 (14.1‐23.9)	427	778,626.9	1.26 (1.10‐1.46)	1.23 (1.06‐1.42)
Q3 (23.9‐35.2)	384	779,380.7	1.20 (1.03‐1.38)	1.13 (0.97‐1.31)
Q4 (35.2‐216.5)	374	766,588.4	1.29 (1.11‐1.49)	1.17 (1.00‐1.36)
p_trend_ c			<0.01	0.11

a Age and sex as strata variables, race as covariate.

b Age and sex as strata variables, race, smoking with pack‐year information, alcohol use, BMI, family history of pancreatic cancer, diabetes, and red meat intake as covariates.

c From a model treating exposure as continuous variable coded as consecutive numbers (eg, 1, 2, 3).

We observed higher pancreatic cancer risk associated with family history of pancreatic cancer (RR 1.97, 95% CI 1.50‐2.58), diabetes (RR 1.32, 95% CI 1.14‐1.54), BMI ≥30 kg/m^2^ (RR 1.25, 95% CI 1.08‐1.46), current smoking (<20 pack‐years: RR 1.43, 95% CI 1.19‐1.73; ≥20 pack‐years: RR 1.76, 95% CI 1.46‐2.12), and red meat (Q4 vs Q1: RR 1.17, 95% CI 1.00‐1.36) (Table 3).

While there was no heterogeneity across race/ethnicity, the associations of these risk factors appeared stronger for particular subgroups (Table 4). The association between first‐degree family history of pancreatic cancer and risk of pancreatic cancer was greatest for Japanese Americans (RR 2.43, 95% CI 1.69‐3.50) and African Americans (RR 2.18, 95% CI 1.07‐4.41), while diabetes had the largest influence in Native Hawaiians (RR 2.01, 95% CI 1.30‐3.12). BMI ≥30 kg/m^2^ had the greatest association for Japanese Americans (RR 1.79, 95% CI 1.30‐2.45), followed by Latino Americans (RR 1.36, 95% CI 0.96‐1.92), and Native Hawaiians (RR 1.28, 95% CI 0.79‐2.08). Current smoking status had the strongest association with pancreatic cancer among Japanese Americans (RR 1.92 95% CI 1.48‐2.49) and African Americans (RR 1.67, 95% CI 1.22‐2.31). Likewise, greater pack‐years of smoking had a larger impact for Japanese Americans (current smoking ≥20 pack‐years: RR 2.28, 95% CI 1.67‐3.12) and African Americans (current smoking ≥20 pack‐years: RR 1.85, 95% CI 1.17‐2.90). Red meat had the strongest influence in African Americans (highest vs lowest quartile: RR 1.48, 95% CI 1.03‐2.14).

**Table 4:** Association between risk factors and pancreatic cancer risk, by race/ethnicity

Risk factor	European American		African American		Native Hawaiian		Japanese American		Latino American		*P* b
	Cases	RR (95% CI)^a^	Cases	RR (95% CI)^a^	Cases	RR (95% CI)^a^	Cases	RR (95% CI)^a^	Cases	RR (95% CI)^a^	
Family history of pancreatic cancer											
No	299	1 (ref)	269	1 (ref)	126	1 (ref)	514	1 (ref)	270	1 (ref)	0.48
Yes	7	1.34 (0.63‐2.85)	8	2.18 (1.07‐4.41)	2	0.93 (0.23‐3.79)	31	2.43 (1.69‐3.50)	6	1.56 (0.69‐3.52)	
Diabetes											
No	284	1 (ref)	231	1 (ref)	100	1 (ref)	482	1 (ref)	219	1 (ref)	0.33
Yes	22	1.40 (0.90‐2.19)	46	1.19 (0.85‐1.65)	28	2.01 (1.30‐3.12)	63	1.10 (0.84‐1.44)	57	1.54 (1.14‐2.09)	
Body mass index (kg/m^2^)											
<25	141	1 (ref)	74	1 (ref)	31	1 (ref)	314	1 (ref)	65	1 (ref)	0.37
25‐30	110	0.91 (0.70‐1.18)	116	0.95 (0.71‐1.28)	52	1.20 (0.76‐1.90)	183	1.17 (0.97‐1.42)	138	1.24 (0.92‐1.68)	
≥30	55	1.06 (0.76‐1.46)	87	0.94 (0.68‐1.29)	45	1.28 (0.79‐2.08)	48	1.79 (1.30‐2.45)	73	1.36 (0.96‐1.92)	
p_trend_ d		0.89		0.72		0.42		<0.01		0.11	
Smoking status^c^											
Never	125	1 (ref)	105	1 (ref)	53	1 (ref)	260	1 (ref)	136	1 (ref)	0.71
Past	124	0.85 (0.66‐1.10)	101	1.08 (0.81‐1.43)	44	0.88 (0.58‐1.33)	199	1.04 (0.85‐1.28)	98	0.93 (0.70‐1.22)	
Current	57	1.39 (1.00‐1.92)	71	1.67 (1.22‐2.31)	31	1.52 (0.95‐2.43)	86	1.92 (1.48‐2.49)	42	1.22 (0.84‐1.75)	
Smoking status, with pack‐year information^c^											
Never	125	1 (ref)	105	1 (ref)	53	1 (ref)	260	1 (ref)	136	1 (ref)	0.71
Past <20 pack‐years	85	0.90 (0.68‐1.19)	72	0.98 (0.72‐1.33)	35	0.98 (0.63‐1.51)	136	1.04 (0.83‐1.30)	86	0.96 (0.72‐1.27)	
Past ≥20 pack‐years	39	0.76 (0.52‐1.10)	29	1.56 (1.01‐2.40)	9	0.62 (0.30‐1.29)	63	1.09 (0.81‐1.48)	12	0.75 (0.41‐1.38)	
Current <20 pack‐years	19	1.29 (0.79‐2.11)	46	1.60 (1.11‐2.31)	15	1.53 (0.84‐2.78)	31	1.53 (1.05‐2.24)	29	1.18 (0.77‐1.78)	
Current ≥20 pack‐years	38	1.43 (0.98‐2.09)	25	1.85 (1.17‐2.90)	16	1.51 (0.84‐2.71)	55	2.28 (1.67‐3.12)	13	1.32 (0.72‐2.42)	
p_trend_ d		0.14		<0.001		0.18		<0.0001		0.52	
Alcohol use (g/day)											
None	103	1 (ref)	170	1 (ref)	67	1 (ref)	347	1 (ref)	133	1 (ref)	0.11
≤24	141	1.02 (0.79‐1.32)	90	0.82 (0.63‐1.07)	44	1.11 (0.75‐1.66)	142	0.91 (0.74‐1.12)	127	1.20 (0.93‐1.55)	
24‐48	34	0.92 (0.62‐1.37)	12	0.75 (0.41‐1.37)	10	1.18 (0.59‐2.37)	41	1.31 (0.93‐1.84)	11	0.70 (0.36‐1.35)	
>48	28	1.29 (0.84‐1.99)	5	0.46 (0.19‐1.15)	7	1.54 (0.68‐3.50)	15	1.01 (0.59‐1.72)	5	0.51 (0.20‐1.25)	
p_trend_ d		0.65		0.04		0.35		0.72		0.44	
Red meat intake (g/kcal/day)											
Q1 (0‐14.1)	88	1 (ref)	54	1 (ref)	27	1 (ref)	130	1 (ref)	48	1 (ref)	0.30
Q2 (14.1‐23.9)	90	1.26 (0.94‐1.70)	66	1.40 (0.97‐2.02)	31	0.80 (0.47‐1.35)	174	1.25 (0.99‐1.57)	66	1.19 (0.82‐1.73)	
Q3 (23.9‐35.2)	66	1.08 (0.78‐1.50)	82	1.78 (1.25‐2.52)	36	0.81 (0.49‐1.36)	135	1.00 (0.78‐1.28)	65	1.10 (0.75‐1.61)	
Q4 (35.2‐216.5)	62	1.22 (0.87‐1.72)	75	1.48 (1.03‐2.14)	34	0.66 (0.39‐1.13)	106	1.03 (0.78‐1.34)	97	1.38 (0.96‐1.98)	
p_trend_ d		0.32		0.02		0.16		0.71		0.08	

a Age and sex as strata variables, race, smoking status with pack‐year information, alcohol use, BMI, family history of pancreatic cancer, diabetes, and red meat intake as covariates.

b *P* values for heterogeneity across race.

c Smoking status and smoking status with pack‐year information assessed in separate models.

d From a model treating exposure as continuous variable coded as consecutive numbers (eg, 1, 2, 3).

From the PAF calculations, diabetes, BMI ≥25, current smoking, and red meat intake altogether accounted for one fifth of pancreatic cancer cases (PAF 21.57, 95% CI 12.37‐29.81). Across racial/ethnic groups, this combination of risk factors explained similar proportions of cases in Latino Americans (32.21, 95% CI 5.94‐51.15), African Americans (29.48, 95% CI 3.73‐48.34), and Japanese Americans (19.75, 95% CI 5.22‐32.06), but lower proportions of cases in European Americans (9.00, 95% CI −12.20 to 26.19) and Native Hawaiians (7.10, 95% CI −46.51 to 41.10). When evaluating the individual risk factors, about 8% of cases could be attributed to BMI ≥25 (PAF 7.59, 95% CI 1.41‐13.37) and 3% to BMI ≥30 (PAF 3.36, 95% CI 0.55‐6.09). These estimates were similar for Japanese Americans (BMI ≥25: PAF 8.63, 95% CI 1.59‐15.16; BMI ≥30 PAF 3.53, 95% CI 0.97‐6.02), but were not statistically significant for the other race/ethnicity subgroups (data not shown). In addition, current smoking explained approximately 4% of cases for the whole cohort (PAF 4.19, 95% CI 1.67‐6.64) and about 6% of cases among Japanese Americans (6.29, 95% CI 2.64‐9.80). However, the PAFs for current smoking were not significant for European Americans (1.46, 95% CI −4.56 to 7.13), African Americans (6.56, 95% CI −0.84 to 13.42), Native Hawaiians (3.37, 95% CI −7.49 to 13.14), or Latino Americans (1.00, 95% CI −4.44 to 6.16). For red meat, about 10% of the cases could be prevented (PAF 9.97, 95% CI 0.99‐18.15) if those in the upper three quartiles of red meat consumption reduced their intake to the first quartile. This decrease was over doubled among African Americans (PAF 26.40, 95% CI 5.73‐42.54), but was not statistically significant for the other populations. There was no decrease in pancreatic cancer burden associated with a modification in diabetes status for the entire cohort (PAF 1.06, 95% CI −1.01 to 3.10) or within any subgroups.

## 4 DISCUSSION

In this study, we evaluated differences in pancreatic cancer incidence and risk factor associations across five racial/ethnic groups in the MEC. We observed higher rates of pancreatic cancer among African Americans, Native Americans, and Japanese Americans, but no difference for Latino Americans, compared to European Americans. While these disparities did not seem to be fully explained by the distribution and effects of risk factors across race/ethnicity, a substantial fraction of pancreatic cancers in our cohort (20%) could be attributed to smoking, adiposity, and red meat intake.

African Americans have had historically higher rates of pancreatic cancer incidence compared to whites.^14^ While risk factors such as obesity, diabetes, and smoking are more prevalent among African Americans, past research has suggested that they do not fully explain the differences in pancreatic cancer incidence.^15^, ^16^ Our findings provide additional support for this disparity, as African Americans had a 20% greater risk of pancreatic cancer compared to European Americans even after adjusting for known risk factors. Thus, other factors not reflected in our analysis may instead account for these differences in incidence. In particular, current evidence has suggested that African Americans may perhaps have higher exposure to carcinogens compared to European Americans. This is supported by research suggesting that African Americans are slower metabolizers of cigarette smoke toxins^17^ and are more likely to have the risky rapid phenotype of the *N*‐acetyltransferase‐1 enzyme,^18^ which promotes the bioactivation of arylamine carcinogens.^19^ Smoking and dietary preferences among this population may also contribute to elevated carcinogen exposure. African Americans tend to smoke menthol cigarettes,^17^, ^20^ which facilitate deeper inhalation and penetration of harmful tobacco products,^21^ and consume higher amounts of well‐done and pan‐fried meats,^18^, ^22^ which have increased levels of heterocyclic amines and polycyclic aromatic hydrocarbons.^23^ These issues could thus potentially explain why the impact of smoking and red meat were stronger for African Americans in our analyses. In addition, higher rates of pancreatic cancer among African Americans may be attributed to differences in genetic susceptibility. As there is currently a lack of studies examining genetics and pancreatic cancer in this population, our results may provide an indication of which specific genetic markers to investigate in future studies.

Pancreatic cancer incidence rates have also been consistently elevated among Native Hawaiians according to the Hawaii Tumor Registry and other SEER registries.^24^, ^25^ In our cohort, Native Hawaiians had the greatest disparity among all race/ethnicity groups, with nearly double the age‐adjusted incidence rate and about a 60% greater relative risk of pancreatic cancer compared to European Americans after risk factor adjustment. This difference in incidence can perhaps be explained by race‐specific variations in the susceptibility and severity of diabetes, which was a strong independent risk factor among Native Hawaiians in our analysis. Literature has shown that there is a disproportionate burden of diabetes among Native Hawaiians, as both the prevalence and risk of diabetes is much higher for Native Hawaiians compared to European Americans.^26^, ^27^, ^28^ There is also evidence suggesting that diabetes may be more critical and advanced in this population. Native Hawaiians are more frequently hospitalized for diabetes^29^ and have a greater likelihood of experiencing diabetes‐related complications and mortality.^26^, ^30^ In addition, Native Hawaiians are more likely to have poorer glycemic control and elevated blood glucose levels,^31^, ^32^ which have been observed to be associated with pancreatic cancer.^33^ Future studies investigating diabetes severity and pancreatic cancer across race/ethnicity groups would be valuable in understanding this increased incidence among Native Hawaiians.

We found that Japanese Americans had both a higher incidence rate and relative risk for pancreatic cancer compared to European Americans. While not previously highlighted, recent incidence data (2009‐2013) from the Hawaii Tumor Registry showed that Japanese individuals had greater age‐adjusted incidence rates compared to European Americans.^24^ We also observed that the relative risk for Japanese Americans remained practically unchanged between our minimally and fully adjusted models, indicating that our covariates did not explain much of the disparity. However, the fact that family history of pancreatic cancer was a stronger risk factor among Japanese Americans than among European Americans suggests that genetics may play a more substantial role in defining risk between race/ethnicity groups. This hypothesis is supported by a previous genome‐wide association study (GWAS) in Japanese individuals, which identified three pancreatic cancer susceptibility loci that were not observed to be associated with risk in prior GWAS studies of individuals of European ancestry.^34^, ^35^, ^36^ A recent GWAS meta‐analysis of three Japanese studies further found a genetic risk marker in *GP2* that is also distinct to this population.^37^ In addition, non‐O blood alleles, which are associated with pancreatic cancer,^38^ appear to be more prevalent in Japanese individuals compared to Europeans.^39^

Our analysis did not detect any difference in pancreatic cancer incidence and risk between Latino Americans and European Americans. Additionally, our race/ethnicity‐stratified models showed that the patterns of associations for each risk factor and pancreatic cancer were similar between European and Latino Americans. Data from SEER and the CDC's National Program of Cancer Registries have reported similar age‐standardized incidence rates between Hispanic and white females, but slightly lower rates for Hispanic men compared to white men.^40^, ^41^ It is possible that the decreased rates for Hispanic males may be attributed to the lower prevalence of smoking in this population.^42^

To our knowledge, this is the first study to calculate PAFs for pancreatic cancer risk factors across race/ethnicity using a formula that accounts for the competing risk of death. Our PAF estimates for current smoking (1%‐7%) are lower than the PAFs for smoking reported in two prior studies (15%‐42%) examining pancreatic cancer risk factors in whites and blacks.^6^, ^16^ However, it may be difficult to make direct comparisons with these studies because the authors used formulas that did not consider follow‐up time or mortality and did not calculate separate PAFs for current smoking alone. In addition, PAF calculations that ignore mortality tend to overestimate the PAF when the follow‐up period is long and the risk factor in question is strongly associated with mortality.^43^ As the average follow‐up time was 16.9 years and smoking had a greater influence on mortality than pancreatic cancer in our cohort, using a formula that addressed censoring due to death was more appropriate and provided a more accurate PAF estimation.

The strengths of our study include the prospective design and large, ethnically diverse population. This permitted us to evaluate disparities in incidence and the influence of risk factors across several race/ethnicity groups, particularly minority populations that have been understudied. Furthermore, calculating PAFs using a formula designed specifically for cohort studies that controlled for competing risk of death and other risk factors provided a more unbiased PAF estimate compared to previous studies. However, as a number of risk factors were assessed by self‐report, the accuracy of some data elements may have been influenced by social desirability bias. Since the baseline questionnaire was administered prior to case identification, we expect any misclassification to be nondifferential and bias our results toward the null. In addition, we used sociocultural categories of race/ethnicity, which does not fully capture all variation in risk associated with genetic ancestry. Incorporating genetic data, as well as biomarker or imaging information to assess pancreatic fat (an emerging risk factor^44^), could have perhaps further accounted for some of the disparities between populations.

This study provided a comprehensive assessment of the incidence and known risk factors for pancreatic cancer across multiple racial/ethnic populations. Our findings suggest that the racial disparities in pancreatic cancer incidence are not fully explained by the interethnic differences in the distribution and effects of these mainly environmental risk factors. Residual risk in pancreatic cancer incidence may in fact be explained by other genetic and biological factors. Thus, future studies should consider more detailed evaluations that incorporate both behavioral and genetic data to further elucidate these discrepancies between race/ethnicity groups.

## Data Availability

Data available on request from the authors.
